# FFF/FDM 3D-Printed Solid Polymer Electrolytes Based on Acrylonitrile Copolymers for Lithium-Ion Batteries

**DOI:** 10.3390/molecules29194526

**Published:** 2024-09-24

**Authors:** Arkadiusz Czerwiński, Magdalena Słojewska, Justyna Jurczak, Maciej Dębowski, Ewa Zygadło-Monikowska

**Affiliations:** Faculty of Chemistry, Warsaw University of Technology, Noakowskiego 3, 00-664 Warsaw, Poland; arkadiusz.czerwinski.dokt@pw.edu.pl (A.C.); magdalena.slojewska.dokt@pw.edu.pl (M.S.); justynaa.jurczak@gmail.com (J.J.); maciej.debowski@pw.edu.pl (M.D.)

**Keywords:** lithium-ion batteries, additive manufacturing, solid polymer electrolytes, fused filament fabrication, poly(AN-*co*-PEGMEA), 3D printing

## Abstract

Lithium-ion batteries (LIBs) are essential in modern electronics, particularly in portable devices and electric vehicles. However, the limited design flexibility of current battery shapes constrains the development of custom-sized power sources for advanced applications like wearable electronics and medical devices. Additive manufacturing (AM), specifically Fused Filament Fabrication (FFF), presents a promising solution by enabling the creation of batteries with customized shapes. This study explores the use of novel poly(acrylonitrile-*co*-polyethylene glycol methyl ether acrylate) (poly(AN-*co*-PEGMEA)) copolymers as solid polymer electrolytes for lithium-ion batteries, optimized for 3D printing using FFF. The copolymers were synthesized with varying AN:PEGMEA ratios, and their physical, thermal, and electrochemical properties were systematically characterized. The study found that a poly(AN-*co*-PEGMEA) 6:1 copolymer ratio offers an optimal balance between printability and ionic conductivity. The successful extrusion of filaments and subsequent 3D printing of complex shapes demonstrate the potential of these materials for next-generation battery designs. The addition of succinonitrile (SCN) as a plasticizer significantly improved ionic conductivity and lithium cation transference numbers, making these copolymers viable for practical applications. This work highlights the potential of combining polymer chemistry with additive manufacturing to provide new opportunities in lithium-ion battery design and function.

## 1. Introduction

Lithium-ion batteries (LIBs) are among the most popular energy storage devices, dominating the market for portable devices and electric vehicles [1,2]. Due to the increasing demand for higher battery capacity and enhanced energy and power densities, significant effort is being devoted to developing new materials for their construction [3,4,5,6,7,8]. In contrast, various types of microelectronics, including medical devices (e.g., biosensors, neurostimulators) and consumer electronics (e.g., wearable electronics such as smart glasses, electronic tattoos, and electronic implants), have different requirements. As these devices rapidly evolve, the shape and size of their batteries become increasingly important. To fully meet user expectations, these devices should be tailored to individual needs, accommodating both ergonomic and aesthetic considerations. This customization is crucial since the design of modern devices is highly valued [9,10,11]. Currently, the shapes of batteries available on the market are primarily limited to various sizes of cuboids and cylinders due to manufacturing constraints [12]. This limitation significantly restricts the design freedom of new electronic devices, which must conform to available battery shapes. A potential solution to this problem is additive manufacturing, which could enable battery shape customization [13,14].

The first successful 3D prints of lithium-ion batteries date back to 2013 [15] when Sun et al. [16] printed a microbattery using the Direct Ink Writing (DIW) technique, which involves applying layers of material in the form of paste, slurry, or ink [17,18]. Since then, interest in this field has grown significantly, with numerous studies published on printed electrodes [19,20,21,22], electrolytes [23,24,25,26,27], and even complete cells [28]. While most research focuses on electrode printing, especially using DIW, there are fewer publications on electrolytes for lithium-ion cells produced through additive manufacturing [29,30,31,32,33]. Yet, electrolytes are a crucial component of batteries and often represent a bottleneck in various battery technologies, including printing [25,34,35,36]. Achieving a true interpenetrating three-dimensional structure of a battery is challenging without a high-quality electrolyte. The electrolyte must meet several requirements, including mechanical strength, effective electrode insulation, high conductivity, and chemical, electrochemical, and thermal stability [37]. Printing a separator and saturating it with a traditional liquid electrolyte could retain current technologies, but this approach compromises safety, which is unacceptable for applications where cells are in close proximity to the human body [38,39].

Polymer electrolytes are promising candidates to replace liquid electrolytes in lithium-ion batteries, offering benefits such as being lightweight, flexible, safe, and moldable [40,41,42]. Gel polymer electrolytes trap volatile organic solvents within their polymer network, reducing the risk of ignition in lithium-ion cells by minimizing leakage and evaporation [43,44]. Solid polymer electrolytes can even eliminate volatile substances entirely, providing a significant safety advantage [45,46,47]. Despite these benefits, polymer electrolytes are not widely used because they generally exhibit lower conductivity compared to liquid electrolytes, making rapid charging and discharging difficult [48]. However, these conductivity levels are acceptable for devices that consume small currents over extended periods and do not require high peak currents [49], such as those needed during the acceleration of electric vehicles. Nevertheless, liquid electrolytes remain prevalent due to the well-developed and cost-effective technology for producing lithium-ion batteries [45]. Combining the advantages of additive manufacturing with polymer electrolytes could be transformative by offering greater design freedom.

Fused Deposition Modeling (FDM)/Fused Filament Fabrication (FFF), which involves melting plastic, appears to be a natural candidate for printing polymer electrolytes. Unlike DIW, FDM/FFF does not require the use of solvents during printing or the optimization of ink rheological properties. The technology of FFF/FDM is well-developed and relatively inexpensive; however, the number of publications on the use of these techniques for lithium-ion battery electrolytes is relatively small. In 2018, Reyes et al. printed cells with a polylactic acid (PLA) separator [28], and in 2019, Maurel et al. used PLA with added SiO_2_ and a plasticizer [50]. In both cases, the printed separators were soaked with a liquid electrolyte. In 2019, Ragones et al. used blends of PLA and PEO with added SiO_2_ or Al_2_O_3_ and lithium salt [51], and Maurel et al. printed electrolytes containing only PEO and lithium salt [52], being among the first to use FDM/FFF to print solid polymer electrolytes for lithium-ion cells. In 2021, Vinegrad and Ragones et al. again used PLA/PEO blends, but this time electrolytes were vacuum-impregnated with an ionic liquid, resulting in increased conductivity compared to pure blends [53].

To our knowledge, no articles have been published so far on the FFF/FDM printing of a true solid polymer electrolyte for lithium-ion cells, where the printing is enabled not by the addition of nanofillers, plasticizers, or printer modifications, but by the use of a copolymer. To achieve this, we synthesized acrylonitrile (AN) copolymers with polyethylene glycol methyl ether acrylate (PEGMEA), from which electrolytes were made by adding various salts with and without functional additives. The electrolytes were extruded into filaments, which were then printed using an unmodified, original 3D printer.

Polyacrylonitrile (PAN) is well-researched for use in lithium-ion cells, mainly as a gel electrolyte (with a conventional organic solvent trapped in its structure) [54,55]. Compared to poly(ethylene oxide) (PEO)-based electrolytes, it offers higher mechanical strength and thermal stability, as well as greater electrochemical oxidation stability. In comparison to poly(vinylidene fluoride) (PVdF)-based or poly(methyl methacrylate) (PMMA)-based electrolytes, PAN also offers better ion transport properties and has the potential to inhibit dendrite growth in lithium batteries [56]. However, despite its many advantages, PAN does not provide sufficient conductivity to be used as a solid polymer electrolyte in mass-produced lithium-ion cells. Still, these properties should be sufficient for printed batteries intended for low-current applications. Unfortunately, extruding PAN is very difficult because its degradation temperature is lower than its melting temperature [57]. While there are ways to address this issue—such as plasticizing the polymer with a solvent like acetonitrile before melting—this process would be highly inefficient and environmentally unfriendly, as simple Fused Filament Fabrication (FFF) requires filament extrusion before printing the desired shape. To resolve this, we copolymerized AN with PEGMEA through radical polymerization. Thanks to the influence of the long side chains of PEGMEA, we were able to significantly lower the glass transition temperature, eliminate crystallization, and reduce the flow temperature, thereby enabling the extrusion of PAN-based copolymers. The oxyethylene groups in PEGMEA also facilitate the dissociation of lithium salts, thus improving ion transport properties. By adjusting the AN ratios, we were able to obtain a material with optimal mechanical properties that allowed for both effective ion transport and successful 3D printing.

## 2. Results and Discussion

### 2.1. Physical Characterization of poly(AN-co-PEGMEA)

#### 2.1.1. Elemental Analysis

The mass percentages of nitrogen (%N), carbon (%C), and hydrogen (%H) in the copolymers with different AN:PEGMEA molar ratios are presented in Table 1.

The nitrogen content (%N) was used to calculate the comonomer ratio, which aligns satisfactorily with the assumed ratio derived from the quantities of monomers used in the reaction. The carbon and nitrogen contents of the copolymers are also within an acceptable range of error, confirming that both monomers have been incorporated into the co-polymer structure in the assumed ratios. The discrepancy between the AN:PEGMEA ratios calculated based on the amounts of monomers used during the reaction and the ratios obtained from elemental analysis results from the uncontrolled radical polymerization mechanism and the effective purification of the products from unreacted monomers.

#### 2.1.2. FTIR Spectroscopy

**Figure 1:**
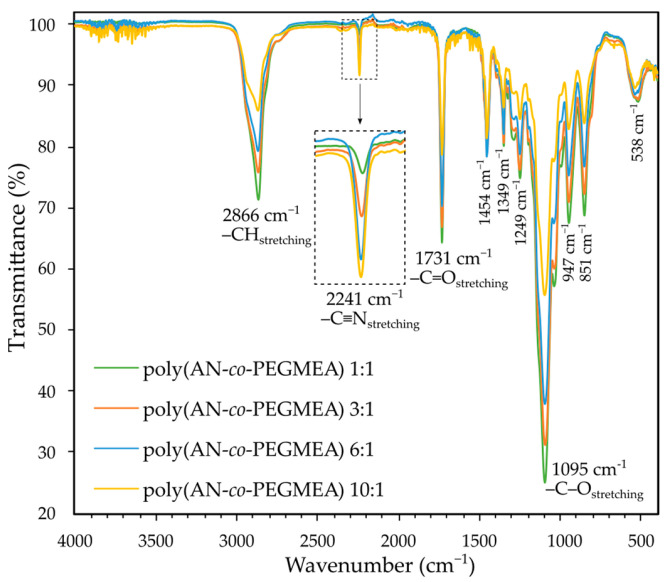
FTIR spectra of poly(AN-*co*-PEGMEA) with AN:PEGMEA ratios of 1:1, 3:1, 6:1, and 10:1.

Figure 1 shows the FTIR spectra of poly(AN-*co*-PEGMEA) copolymers with AN:PEGMEA ratios of 1:1, 3:1, 6:1, and 10:1. These spectra confirm the formation of compounds with varying proportions of monomers in the structure. In the region of 2241 cm^−1^, a characteristic band for acrylonitrile is observed, originating from the stretching vibrations of the nitrile group. The intensity of this band varies depending on the AN:PEGMEA ratio, being lowest for a ratio of 1:1 and highest for a ratio of 10:1. Conversely, the situation is reversed for the bands at 1731 cm^−1^ and 1095 cm^−1^, corresponding to the stretching vibrations of the C=O and C-O ester groups, respectively. The intensity of these bands is greatest when PEGMEA has the highest contribution to the polymer structure. The same trend is observed for the band at 2866 cm^−1^, corresponding to the stretching vibrations of -CH, as well as for the two bands at 851 cm^−1^ and 947 cm^−1^, which are characteristic of PEGMEA. The disappearance of bands corresponding to double bonds present exclusively in the monomers also confirms the formation of polymeric compounds and the successful purification of the product from residual unreacted substances. The FTIR curves of the monomers are presented in Figures S1 and S2.

#### 2.1.3. ^1^H NMR Spectroscopy

**Figure 2:**
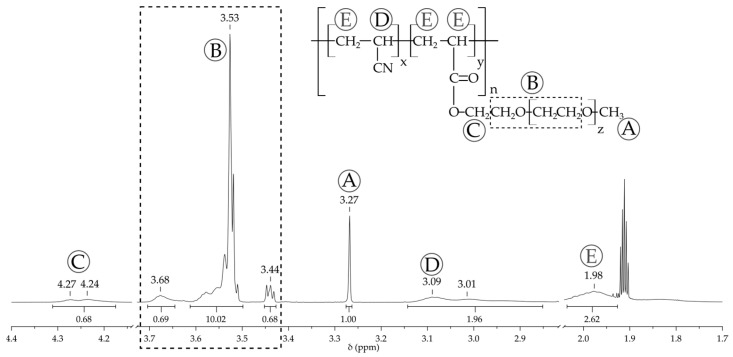
^1^H NMR spectra of the poly(AN-*co*-PEGMEA) with an AN:PEGMEA ratio of 6:1.

Figure 2 illustrates the ^1^H NMR spectra of the poly(AN-*co*-PEGMEA) with an AN:PEGMEA ratio of 6:1.

The obtained ^1^H NMR spectra confirm the formation of the poly(AN-*co*-PEGMEA). The spectrum displays signals originating from protons in the copolymer’s side chains: a sharp singlet from the methoxy group (-O-CH_3_) at 3.27 ppm (A); an intense, sharp signal with a broad base from the oxyethylene chain at 3.53 ppm (B); and a signal from the -CH_2_- group adjacent to the ester group at 4.27 ppm (C). Signals from the copolymer’s main chain (-CH_2_-CH-) are visible as a broad peak around 1.98 ppm (E), except for the protons of the -CH(CN)- group (D), which appear as a very broad peak with a maximum at 3.09 ppm. After integration (using the methoxy group -OCH_3_ signal as the normalization basis), the average length of the oxyethylene chain is calculated as z ≈ 8, which corresponds to a molar mass of the PEGMEA monomer consistent with the manufacturer’s declared value of 480 g/mol. The ratio of signals labeled D and A allows for the calculation of the AN:PEGMEA ratio, presented in Table 1, which is consistent with the results obtained from elemental analysis.

#### 2.1.4. Differential Scanning Calorimetry (DSC)

**Figure 3:**
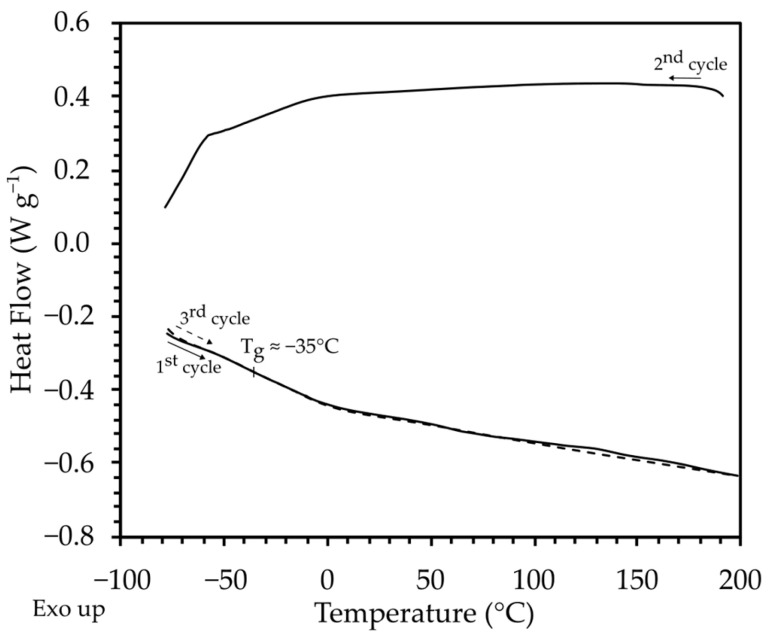
DSC thermogram of poly(AN-co-PEGMEA) 6:1.

The results of differential scanning calorimetry (DSC) measurements for the poly(AN-*co*-PEGMEA) 6:1 are shown in Figure 3. The copolymers exhibit complete amorphousness, with no signals corresponding to crystallization or melting phases observed across the entire tested range. The glass transition temperature is approximately −35 °C. These properties are highly advantageous for using the copolymer as a matrix in solid polymer electrolytes.

### 2.2. Electrochemical Characterization of poly(AN-co-PEGMEA) Electrolytes

#### 2.2.1. Ionic Conductivity

**Figure 4:**
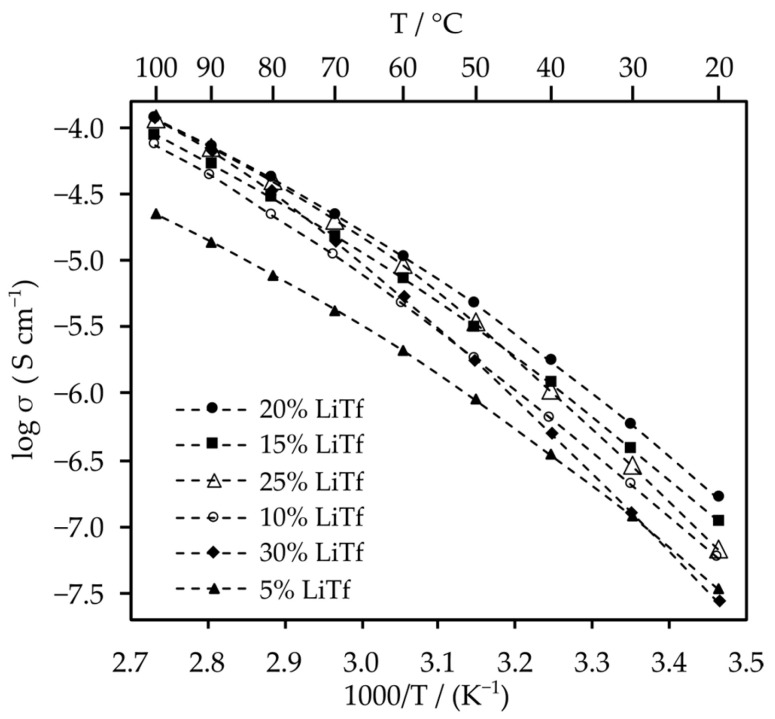
Dependence of ionic conductivity on temperature for electrolytes with varying concentrations of CF_3_SO_3_Li in a poly(AN-*co*-PEGMEA) 3:1 matrix. The dashed lines are guidelines for the eye. The EIS plots are presented in Figure S3.

Solid polymer electrolytes based on poly(AN-*co*-PEGMEA) copolymers and CF_3_SO_3_Li (LiTf) lithium salt were examined using electrochemical impedance spectroscopy (EIS). Figure 4 presents the logarithmic relationship of ionic conductivity as a function of the inverse temperature for varying concentrations of LiTf in a poly(AN-*co*-PEGMEA) 3:1 matrix.

The highest ionic conductivity across the entire temperature range is observed for the 20 wt.% salt concentration, reaching values of 1.6 × 10^−7^ S/cm at 20 °C, 1.1 × 10^−5^ S/cm at 60 °C, and 1.2 × 10^−4^ S/cm at 100 °C. The concentrations of 5, 10, 15, and 20 wt.% exhibit similar slopes in their curves, indicating comparable activation energies. In contrast, the slopes for the 25 and 30 wt.% concentrations are significantly steeper, intersecting with the other concentrations and converging near 100 °C. This behavior is likely due to the formation of aggregates and increased viscosity, which hinder ion movement within the system. The ionic conductivity reaches similar values for the 5 wt.% and 30 wt.% concentrations at 30 °C, whereas at 20 °C, the 30 wt.% concentration has the lowest value of all.

**Figure 5:**
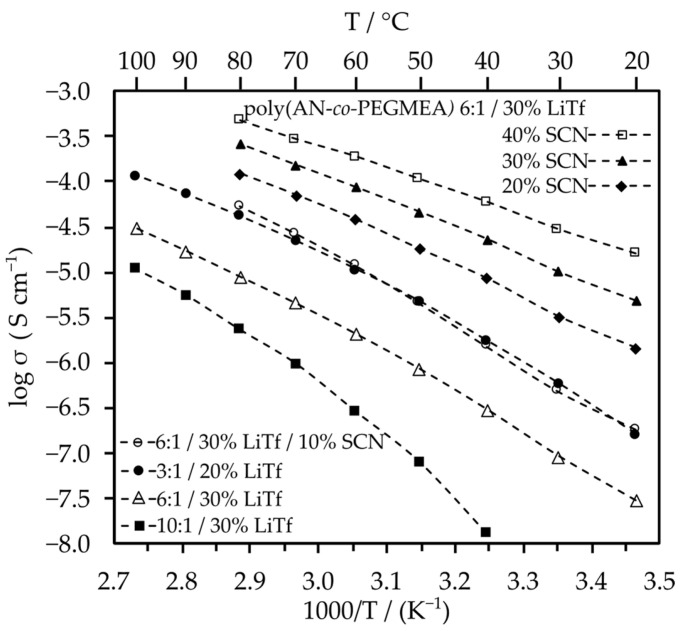
Dependence of ionic conductivity on temperature for electrolytes with addition of SCN plasticizer. The dashed lines are guidelines for the eye. The EIS plots are presented in Figure S4.

To increase the ionic conductivity of the electrolytes while remaining in the category of solid-state polymer electrolytes, we decided to add a solid plasticizer to facilitate the segmental motion of polymer chains. For this purpose, we introduced succinonitrile (SCN) into the poly(AN-*co*-PEGMEA) 6:1 matrix. The effect of the plasticizer addition on ionic conductivity is shown in Figure 5. The pure LiTf electrolyte with the poly(AN-*co*-PEGMEA) 6:1 matrix exhibited a conductivity of 2.6 × 10^−8^ S/cm at 20 °C and 1.4 × 10^−5^ S/cm at 80 °C. The addition of 40% SCN increased the conductivity to 1.6 × 10^−5^ S/cm and 4.0 × 10^−4^ S/cm at 20 °C and 80 °C, respectively. Interestingly, very similar conductivities across the entire studied range were obtained for electrolytes with the poly(AN-*co*-PEGMEA) 3:1 matrix without the addition of a plasticizer and for electrolytes with the poly(AN-*co*-PEGMEA) 6:1 matrix with the addition of 10% SCN. This indicates that ionic conductivity can be controlled either by changing the ratio of monomers in the copolymer or by plasticizing the material with low molecular weight compounds. For comparison, the plot also includes the electrolyte with the poly(AN-*co*-PEGMEA) 10:1 matrix, which shows the lowest conductivity values. The poly(AN-*co*-PEGMEA) 1:1 copolymer was not studied because it was too viscous and did not hold promise for use in 3D printing by the FDM method.

#### 2.2.2. Lithium Cation Transference Number

The lithium cation transference numbers were investigated using the Bruce–Vincent polarization method. Electrolytes with the poly(AN-*co*-PEGMEA) 3:1 or 6:1 matrix and without a plasticizer exhibited typical values for solid polymer electrolytes with oxyethylene chains, around 0.2. However, the addition of SCN increased the transference numbers to values above 0.7. Detailed data are presented in Table 2. This increase can be explained by the favorable interaction of succinonitrile with lithium cations, which weakens the coordination of cations by oxyethylene groups, thus facilitating their transport in the electrolyte.

#### 2.2.3. Cyclic Voltammetry

**Figure 6:**
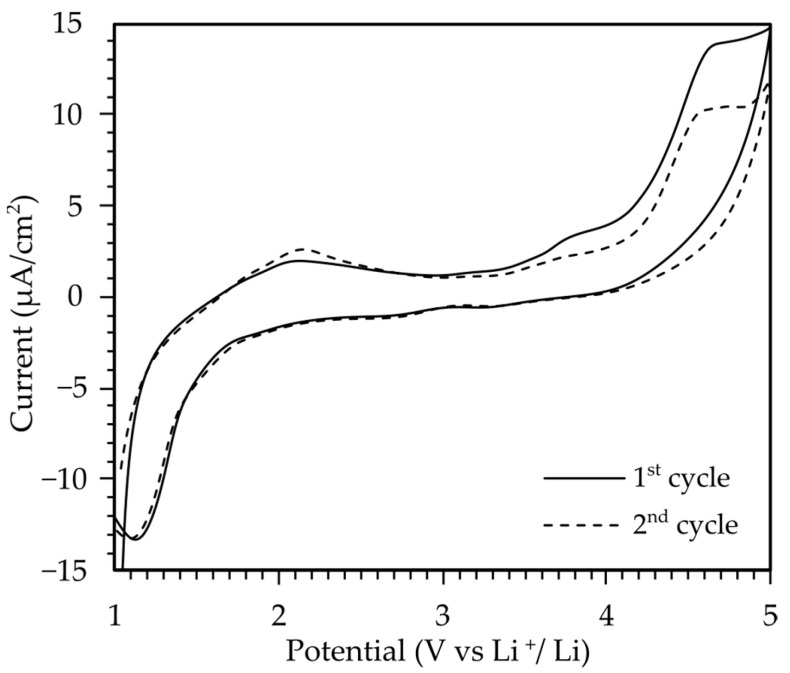
Cyclic voltammetry curves of poly(AN-*co*-PEGMEA) 6:1 + 20% LiTf.

Cyclic voltammetry (CV) was used to investigate the electrochemical stability of the poly(AN-*co*-PEGMEA) 6:1 electrolyte within the voltage range of 0–5 V vs. Li/Li^+^ at a scanning rate of 1 mV/s (Figure 6). During the first cycle scan, an increase in oxidation current density from approximately 4.0 V to 5.0 V was observed, which could be attributed to the oxidation of oxyethylene groups in the copolymer’s side chains. After switching the polarization, the current density remained stable until approximately 2 V, where reduction processes became visible, and increased more sharply around 1.5 V, suggesting lithium stripping from the membrane and its deposition on stainless steel (SS). After the second polarization switch, the lithium oxidation process was no longer visible, indicating irreversible lithium extraction from the polymer membrane. Based on the cyclic voltammetry analysis, the electrochemical stability window of the examined polymers is in the range of 1.7 V to 4.0 V relative to lithium. Above this value, the electrolyte begins to decompose.

#### 2.2.4. TOF-SIMS and EDS Spectroscopy

**Figure 7:**
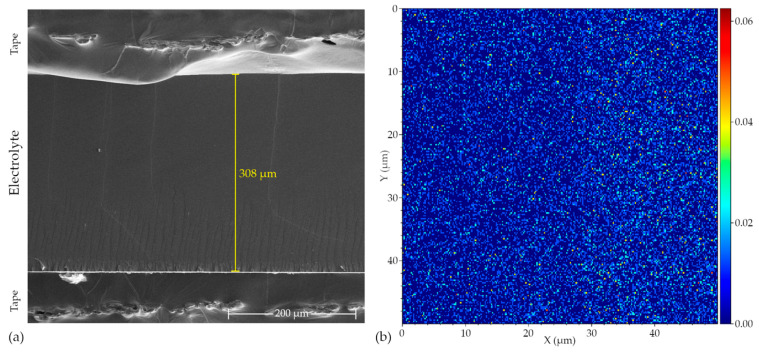
(**a**) SEM image of the cross-section of poly(AN-co-PEGMEA) 10:1 with 20% LiTf; (**b**) TOF-SIMS analysis showing the lithium distribution across the cross-section of poly(AN-co-PEGMEA) 10:1 with 20% LiTf.

Poly(AN-co-PEGMEA) electrolytes were analyzed using Time-of-Flight Secondary Ion Mass Spectrometry (TOF-SIMS) and Energy-Dispersive X-ray Spectroscopy (EDS). Figure 7a shows a Scanning Electron Microscopy (SEM) image of the cross-section of poly(AN-co-PEGMEA) 10:1 with 20% LiTf, while Figure 7b and Figure S5 presents the lithium distribution analyzed by TOF-SIMS. The electrolyte thickness measured from the SEM images aligns well with the results obtained using a micrometer during conductivity testing and is approximately 308 µm. The sample cross-section is uniform, with no visible agglomerates or undissolved or crystallized salt. TOF-SIMS also confirms the uniform distribution of the salt throughout the entire volume of the electrolyte, which is consistent with the EDS results for sulfur and fluorine (Figures S6 and S7). This indicates good compatibility between the poly(AN-co-PEGMEA) polymer matrix and the lithium salt.

### 2.3. 3D Printing of poly(AN-co-PEGMEA)

The feasibility of printing poly(AN-*co*-PEGMEA) using the FFF/FDM technique was investigated. The copolymer with a 1:1 AN:PEGMEA ratio was highly viscous, resembling a thick liquid rather than a solid, which made it impossible to produce a filament. Therefore, it was excluded from further testing. Filament extrusion tests were conducted with the remaining copolymers using a twin-screw extruder. Extruding filament from the 3:1 copolymer proved to be a significant challenge due to its increased adhesion and cohesion properties, and the extruder’s feed design prevented the extrusion of homogeneous filaments longer than 20 cm, which was insufficient for printing. Filaments with a diameter of approximately 1.75 mm were successfully extruded from copolymers with ratios of 6:1 and 10:1 at temperatures of 170 °C and 190 °C, respectively. The filament from the 6:1 copolymer was significantly more flexible than the 10:1 copolymer, even more so than most commercial TPU (Thermoplastic Polyurethane) filaments, yet it was homogeneous and free of air cavities. Considering the higher electrochemical conductivity of the copolymer with a lower acrylonitrile content, along with its elasticity and increased adhesion—which are highly beneficial for electrode interaction—the poly(AN-*co*-PEGMEA) 6:1 copolymer was selected for most of the printing tests.

**Figure 8:**
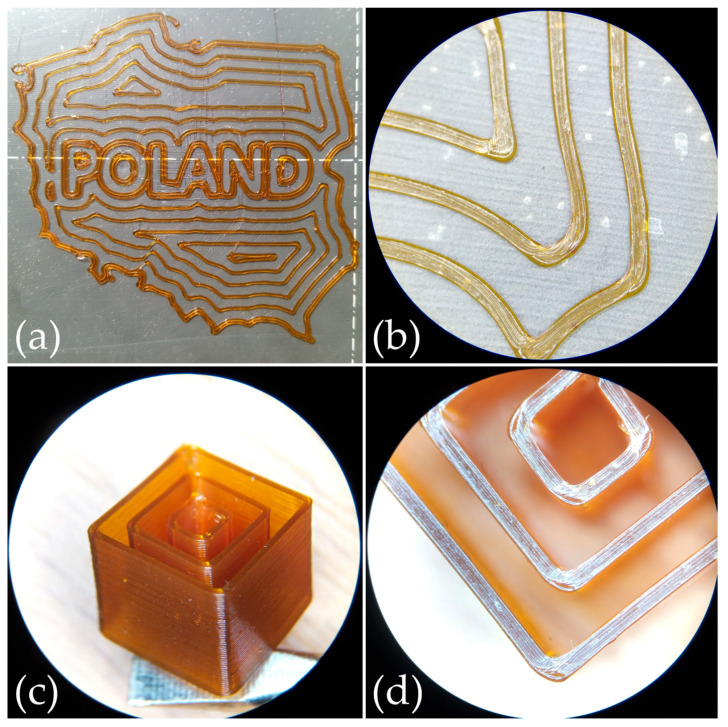
poly(AN-*co*-PEGMEA) 6:1 FFF/FDM 3D prints: (**a**) Print of the first two layers outlining the shape of Poland with a concentric fill pattern. (**b**) Close-up of the individual paths and corners in the first layer. (**c**) Printed concentric cube with an outer edge length of 10 mm. (**d**) Top-down view of the printed cube.

The main idea behind creating these copolymers was to find an optimal balance between electrochemical properties and printability, in order to propose a material that could be printed using simple, widely available 3D printers without requiring significant hardware modifications. Prints were made using a commercial 3D printer, the Prusa i3 MK3S+. Due to the high flexibility of the filament, the filament sensor, which in this version of the printer consists of a ball that introduced additional resistance, was disconnected. Apart from that, the printer was not modified in any way. The print tests began by evaluating shape accuracy. The minimum printing temperature for the 6:1 copolymer was determined to be 150 °C, with the bed temperature set at 50 °C. Figure 8a shows the print of the first two layers outlining the shape of the territory of Poland with a concentric fill pattern. High-quality prints were achieved without visible artifacts, particularly without stringing (oozing). Figure 8b shows a close-up of the individual paths and corners for the first layer of that shape. Using a 0.4 mm nozzle, fairly uniform paths were obtained with a width very close to the nozzle diameter. Next, a multi-layer printing test of single-outline walls was conducted. Figure 8c shows a printed concentric cube with an outer edge length of 10 mm, and Figure 8d shows a top-down view of that print. High-quality, partially transparent prints with a uniform layer distribution and accurate shape reproduction were obtained. It is also noteworthy that the prints did not require post-processing, which could be significant when printing an entire gyroid-structured battery. In such a case, the electrolyte should be applied frequently and in multiple locations alternately with the electrodes, leaving no room for additional processing.

**Figure 9:**
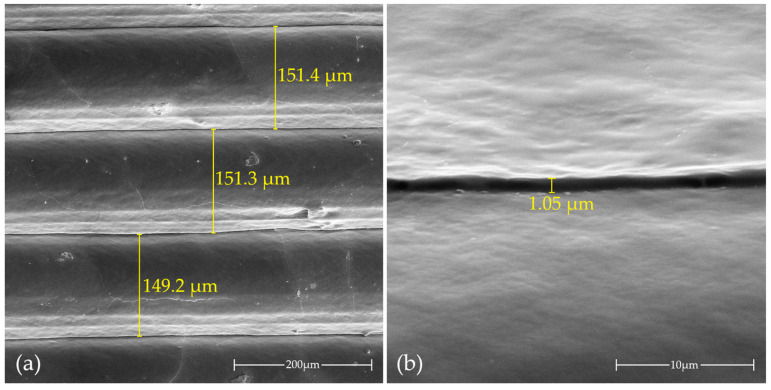
SEM images of a section of the outer wall of an FFF/FDM 3D-printed concentric cube from the poly(AN-co-PEGMEA) 6:1 copolymer: (**a**) view of several layers; (**b**) close-up of the interlayer gap.

The print quality was examined using a SEM. Figure 9 presents an SEM image of a section of the outer wall of the previously described printed cuboid. In Figure 9a, the print layers are shown to be evenly distributed, with a height closely matching the intended 150 µm. However, when considering the broader challenge of battery printing using additive techniques, it is important to note that one of the key characteristics of most 3D printing methods—layering—may have a significant negative impact on cell durability. Figure 9b provides a close-up of the interlayer gap in the obtained print, which measures approximately 1 µm in height. It is well known that, while relatively high-quality prints may appear airtight on a macroscopic scale, they often are not, leading to issues such as liquid leakage when a printed object is filled. This problem arises from insufficient bonding between layers at a microscopic level. In the case of the material we have proposed, which could serve as a solid polymer electrolyte in fully printed cells, this issue may be less critical since no liquid is involved. However, printing a complete cell also necessitates the printing of electrodes. Interlayer spaces might reduce the electron conduction surface area, thereby diminishing cell performance at higher currents. Additionally, printed enclosures may not be sufficiently airtight, potentially allowing moisture and oxygen ingress, which are detrimental to lithium-ion cells, especially lithium cells. Another approach to creating a printed cell is to introduce an electrode slurry between the printed electrolyte. In such a scenario, the gap size must be smaller than the smallest electrode particles to prevent them from penetrating during impregnation into adjacent layers, which could result in mixing and short-circuiting of cathode and anode materials, leading to cell failure.

There are methods to improve layer bonding, such as increasing the printing temperature, but this can cause stringing, which may degrade print quality in polymer electrolytes and could potentially lead to short circuits when printing electrodes. Additionally, print inhomogeneities may occur locally and can be equally hazardous. Figure S8 illustrates an SEM image of a print from the same copolymer at the minimum printing temperature of 150 °C. Although the print quality appears good on a macroscopic scale, with evenly distributed layers in most areas, significant local delamination is observed in one region, which could result in cell failure in certain circumstances.

## 3. Materials and Methods

### 3.1. Chemicals and Reagents

Acrylonitrile (AN, 99%), poly(ethylene glycol) methyl ether acrylate (PEGMEA, average Mn = 480 g/mol), and acetonitrile (99%, anhydrous) were obtained from Sigma-Aldrich (St. Louis, MO, USA) and stored over molecular sieves under an argon atmosphere. Prior to polymerization, the monomers were purified by passing them through a column packed with activated basic aluminum oxide (Sigma-Aldrich) to remove inhibitors. Azobisisobutyronitrile (AIBN, 98%) was also sourced from Sigma-Aldrich and was purified by crystallization from methanol and stored in a freezer. Lithium trifluoromethanesulfonate (LiTf, 96%) was procured from Sigma-Aldrich, dried under reduced pressure at 130 °C, and kept under argon. Metallic lithium, in ribbon form (1.5 mm thick and 100 mm wide), was obtained from Sigma-Aldrich. Deuterated acetonitrile (CD_3_CN, ARMAR Chemicals, Cunnersdorf, Germany) was stored over molecular sieves in a refrigerator under an inert gas atmosphere. Diethyl ether (POCh S.A., Gliwice, Poland) was used as received.

### 3.2. Synthesis of Poly(AN-co-PEGMEA)

In a 50 mL glass ampoule reactor equipped with a magnetic stirring element, 0.100 g of AIBN was added. The reactor was sealed and subjected to three cycles of vacuum-argon treatment. Approximately 10 cm^3^ of acetonitrile was introduced as a solvent, followed by the addition of 2.68 g of acrylonitrile and 4.03 g of polyethylene glycol methyl ether acrylate (PEGMEA), both previously purified by passing through alumina. The molar ratio of AN to PEGMEA was maintained at 6:1. The reactor was mixed thoroughly and placed in a laboratory oven at 60 °C for 6 h. During the reaction, the viscosity of the mixture increased, and a yellowish coloration was observed. Subsequently, the reactor was vented using a needle, opened, and its contents were poured into approximately 150 mL of diethyl ether cooled in a dry ice bath to precipitate the copolymer. The reactor was rinsed with a small amount of acetonitrile, and the product was extracted with ether by intensive mixing and kneading in a beaker. This dissolution and precipitation process was repeated three times to purify the product, which was then transferred to a crystallization vessel. All procedures were conducted under fume hoods in an ambient air atmosphere. The product was subsequently dried in a vacuum oven at 50 °C for 72 h. After this period, the polymer was transferred to a glove box. An analogous procedure was applied for the synthesis of the remaining copolymers, varying only the monomer ratios, specifically AN:PEGMEA ratios of 1:1, 3:1, and 10:1. The overall yield of the entire process, including the multiple precipitation steps, ranged from 78% to 86%.

### 3.3. Preparation of Electrolytes

The electrolytes were obtained using the solvent-casting method. In a previously argon-flushed 50 mL round-bottom flask, the appropriate copolymer, lithium salt, and, if necessary, a plasticizer (SCN) were added to achieve a total mass of 1 g. The mixture was then dissolved in 10 cm^3^ of solvent and poured onto Teflon petri dishes with an inner diameter of 50 mm, which were placed in an argon-filled vacuum oven. The electrolytes were dried under dynamic vacuum conditions by gradually reducing the pressure and subsequently backfilling with argon in a continuous cycle. Upon reaching full vacuum achieved by an oil pump (10^−3^ mbar), the electrolytes were dried at room temperature for 24 h, followed by 48 h at 50 °C. The process yielded homogeneous, partially transparent films from all copolymers, except for the AN:PEGMEA ratio of 1:1, which exhibited intermediate properties between a gel and a solid.

### 3.4. Experimental Techniques

Elemental analysis (EA) was performed on a CHNS Analyzer VARIO EL III (Elemen-tar Analysensysteme GmbH, Langenselbold, Germany).

The chemical structure of copolymers was characterized by ^1^H spectroscopy in solutions in CD_3_CN (JEOL JNM-ECZL 600 MHz (JEOL (EUROPE) SAS, Croissy-sur-Seine, France)).

FTIR spectra were obtained by 16 scans with a resolution of 0.1 cm on a Nicolet 6700 FTIR spectrometer (Thermo Fisher Scientific, Dreieich, Germany) with the use of the ATR technique.

Differential scanning calorimetry (DSC) was used to assess the type and quality of phase transformations occurring in the tested materials. Measurements were carried out on a Q2000 (TA Instruments, New Castle, DE, USA) differential scanning calorimeter in a −80 to 200 °C temperature range with a heating/cooling rate of 10 °C/min in hermetically closed aluminum vessels. All samples were prepared in an Ar-filled glovebox.

Ionic conductivity of the electrolytes was determined via impedance measurements using a VSP–3e potentiostat (Bio-Logic, Seyssinet-Pariset, France) within a frequency range of 800 kHz to 1 Hz and a temperature range of 20 to 100 °C. The samples were stored in symmetrical cells with stainless steel electrodes. Ionic conductivity was calculated according to the following equation:

σ = l/(S × R)(1)

where R represents the resistance determined from the impedance measurement, S is the surface area of the electrolyte, and l is the thickness of the electrolyte.

The transference number (t_+_) of lithium cations was determined using the polarization method with the Bruce–Vincent correction. According to this method, the electrolyte was placed in a symmetrical Li|electrolyte|Li system at a temperature of 60 °C. A constant bias voltage of 20 mV was applied to the system. Then, the system was monitored until the polarization current reached a steady state (I_ss_ value). Additionally, impedance spectra were recorded before and after polarization. The following equation was used to calculate the transference number:

t_+_ = I_ss_ (U − I_0_R_0_)/[I_0_ (U − I_ss_R_ss_)] (2)

where U represents applied polarization potential, I_0_ and I_SS_ represent the initial and steady-state currents, respectively, and R_0_ and R_SS_ represent the corresponding initial and steady-state resistances of the solid-state interface calculated from the impedance plots before and after polarization.

Cyclic voltammetry (CV) was conducted in a two-electrode Swagelok-type system (Claio, Poznań, Poland). The system included a stainless steel electrode as the working electrode (WE) and a lithium electrode as the counter electrode (CE) and as the reference electrode (RE). The measurements were carried out at a scanning speed of 1 mV/s within a voltage range of 0–5 V vs. Li/Li^+^. The measurements were performed at a temperature of 60 °C.

Three-dimensional printing of the copolymers was conducted using a Prusa i3 MK3S+ printer (Prusa Research, Prague, Czech Republic) without the filament sensor. The printer was otherwise unmodified, operating with a standard 0.4 mm nozzle. Models were prepared using Fusion 360 and sliced with a Prusa Slicer.

Filaments were extruded using a Haake Minilab II (Thermo Fisher Scientific, Karlsruhe, Germany) extruder equipped with a co-rotating screw and a 2.0 mm round die. The filament was wound onto an empty spool driven by a stepper motor powered by an Arduino Ramps 1.4. Filament diameter was measured using a micrometer screw gauge with an accuracy of 0.01 mm.

SEM images of the samples were taken using a dual beam Helios 5 PFIB scanning electron microscope (ThermoFisher Scientific, Hillsboro, OR, USA) equipped with a high-resolution Elstar field emission electron column, a Phoenix ion column with inductively coupled Xe+ plasma, and a set of Elstar detectors for secondary electrons: standard ETD detector placed in the chamber and intra-column TLD detector for high-resolution immersion imaging. The working distance of ca. 4.0 mm. For SEM imaging the accelerating voltage of the electron beam of 5 kV were used. All samples were sputtered with a 10 nm-thick carbon electron conductive layer using a CCU-010 high-vacuum sputtering machine (Safematic GmbH, Zizers, Switzerland). Mappings of the samples by means of Energy Dispersive Spectroscopy (EDS) or Time-of-Flight Secondary Ion Mass Spectrometry (TOF-SIMS) measurements were conducted on the same SEM apparatus with accelerating voltage of the electron beam of 10 kV. 

## 4. Conclusions

In this study, we propose the use of copolymers as a method for tailoring the electrochemical and mechanical properties of materials by adjusting the monomer ratios, thereby enabling the 3D printing of solid polymer electrolytes using FFF/FDM techniques. To achieve this, poly(acrylonitrile-co-polyethylene glycol methyl ether acrylate) (p(AN-co-PEGMEA)) with various AN:PEGMEA ratios were successfully synthesized, used to create electrolytes, and then 3D printed. By using these copolymers, we have enabled the extrusion and printing of polymers containing acrylonitrile. Printing the AN (PAN) homopolymer by FFF/FDM is usually difficult, or even impossible, because its melting temperature is higher than its decomposition temperature.

Through systematic synthesis and characterization of poly(AN-co-PEGMEA), we identified the 6:1 ratio as optimal for achieving a balance between mechanical properties and ionic conductivity. The higher the AN content, the more rigid, less adhesive, and cohesive the material becomes, which facilitates 3D printing and filament extrusion. The copolymers exhibited amorphous structures, favorable glass transition temperatures, and acceptable ionic conductivities suitable for low-power electronic devices. The introduction of succinonitrile (SCN) as a plasticizer further enhanced the electrolytes’ ionic conductivity and lithium cation transference number, highlighting its role in facilitating ion transport.

The presented studies demonstrate that the use of acrylonitrile copolymers with PEGMEA enables successful extrusion and 3D printing into complex shapes using a standard, unmodified commercial 3D printer. This marks a significant step forward in the development of customizable lithium-ion batteries, offering greater design freedom for wearable electronics, medical devices, and other specialized applications. Future research should focus on optimizing the electrolyte formulations for higher conductivity and exploring their integration into full cell architectures to validate their performance in practical applications.

## Figures and Tables

**Table 1:** Elemental analysis of nitrogen (%N), carbon (%C), and hydrogen (%H) in the copolymers with varying AN:PEGMEA molar ratios. %C_calculated_ and %H_calculated_ refer to the theoretical content of these elements based on %N. The AN:PEGMEA ratio determined from ^1^H NMR is discussed in Section 2.1.3.

Assumed AN:PEGMEA Ratio	%N	%C	%H	Calculated AN:PEGMEA Ratio (%N)	%C Calculated	%H Calculated	^1^H NMR AN:PEGMEA Ratio
1.01	3.105	55.865	8.705	1.21	56.32	8.35	1.11
3.01	6.305	52.285	7.255	2.84	57.91	7.98	2.85
6.02	10.355	60.085	8.110	5.84	59.93	7.52	5.88
10.08	13.510	60.145	7.460	9.48	61.50	7.15	9.51

**Table 2:** Values of the lithium cation transference numbers of poly(AN-*co*-PEGMEA) electrolytes.

	t_+_
poly(AN-*co*-PEGMEA) 3:1/30wt.% LiTf	0.29
poly(AN-*co*-PEGMEA) 6:1/30wt.% LiTf	0.16
poly(AN-*co*-PEGMEA) 6:1/30wt.% LiTf/10wt.% SCN	0.73
poly(AN-*co*-PEGMEA) 6:1/30wt.% LiTf/20wt.% SCN	0.85
poly(AN-*co*-PEGMEA) 6:1/30wt.% LiTf/30wt.% SCN	0.69
poly(AN-*co*-PEGMEA) 6:1/30wt.% LiTf/40wt.% SCN	0.71

## Data Availability

Data can be made available upon reasonable request.

## Supplementary Materials

The following supporting information can be downloaded at https://www.mdpi.com/article/10.3390/molecules29194526/s1: Figure S1, FTIR spectrum of AN; Figure S2, FTIR spectrum of PEGMEA; Figure S3, EIS plots of poly(AN-co-PEGMEA) 3:1 electrolytes with various CF_3_SO_3_Li concentrations at 30 °C; Figure S4, EIS plots of poly(AN-co-PEGMEA) electrolytes with addition of SCN plasticizer at 30 °C; Figure S5, TOF-SIMS lithium distribution data of poly(AN-co-PEGMEA) 10:1 with 20% LiTf; Figure S6, EDS sulfur distribution of poly(AN-co-PEGMEA) 10:1 with 20% LiTf; Figure S7, EDS sulfur distribution of poly(AN-co-PEGMEA) 10:1 with 20% LiTf; Figure S8, SEM image of a section of the outer wall of an FFF/FDM 3D-printed concentric cube made from a poly(AN-co-PEGMEA) 6:1 copolymer and printed at a temperature of 150 °C, which was determined to be the minimum extrusion temperature.
