# Supplementary figures and images for: FFF/FDM 3D-Printed Solid Polymer Electrolytes Based on Acrylonitrile Copolymers for Lithium-Ion Batteries
Source: Molecules. 2024 Sep 24;29(19):4526. doi: 10.3390/molecules29194526 (PMC11477558; doi:10.3390/molecules29194526)

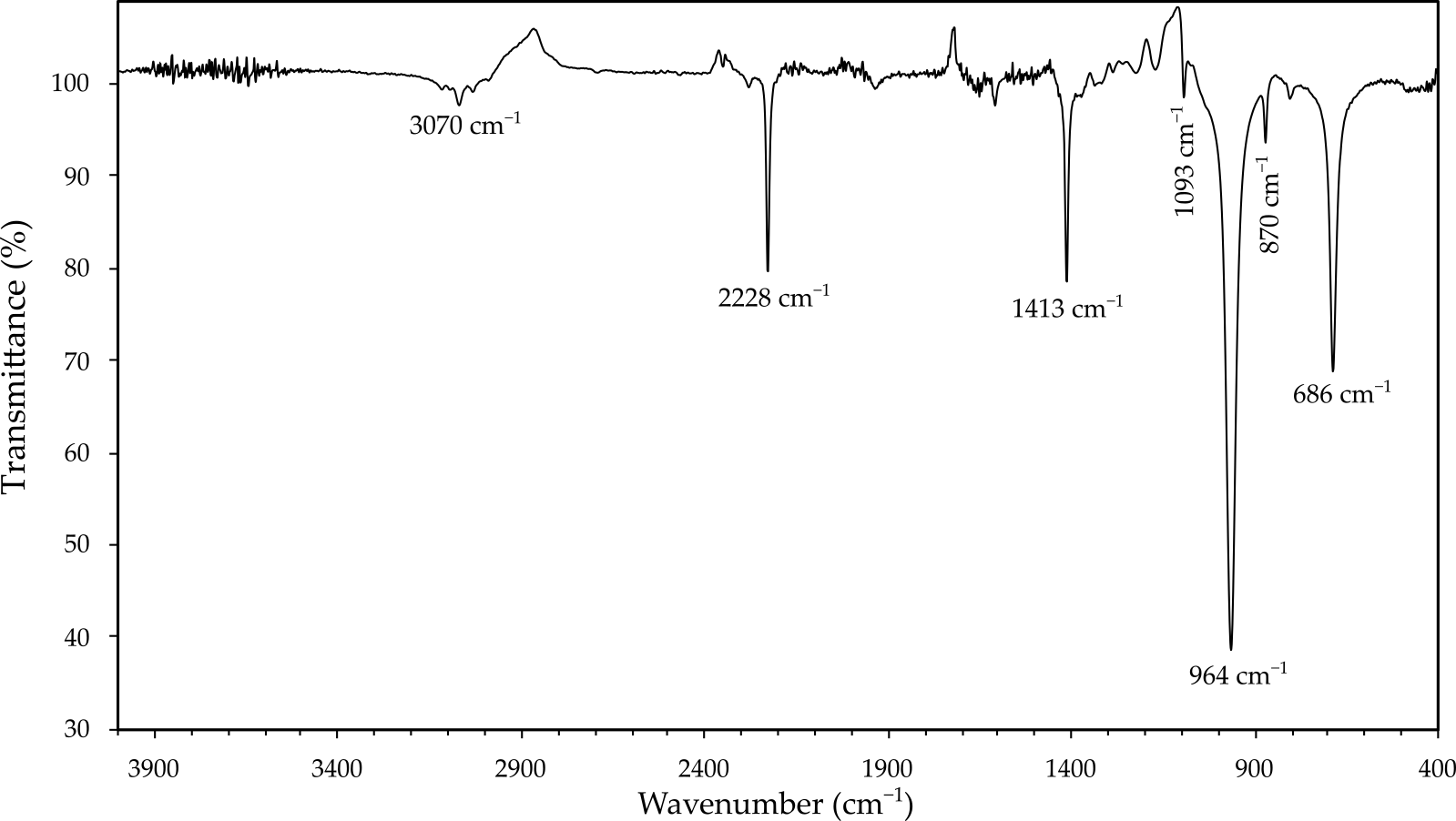

Supplement: Supplementary file 1 [file molecules-29-04526-s001.zip › molecules-3198227-supplementary/Figure S1.tiff]

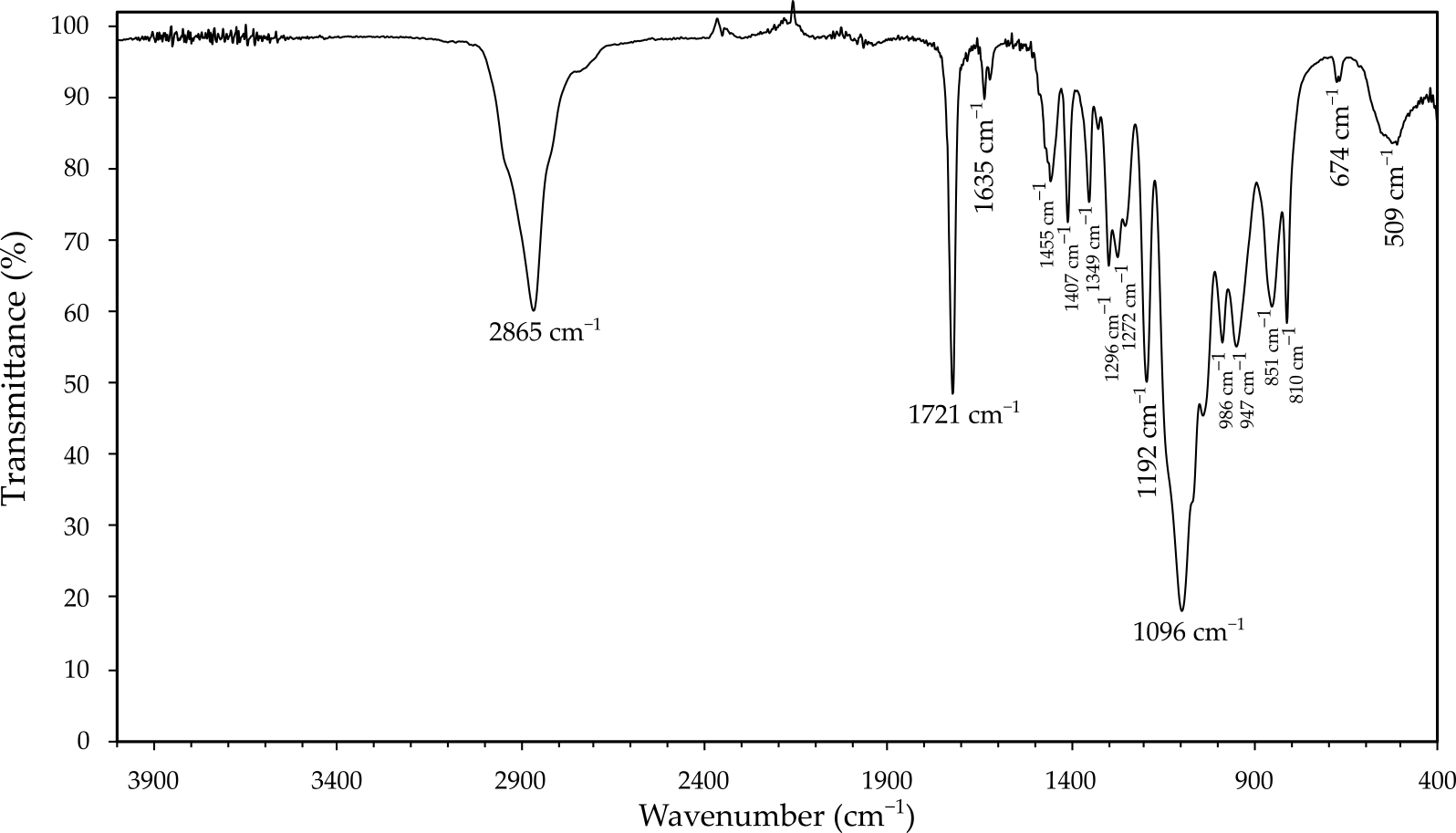

Supplement: Supplementary file 1 [file molecules-29-04526-s001.zip › molecules-3198227-supplementary/Figure S2.tiff]

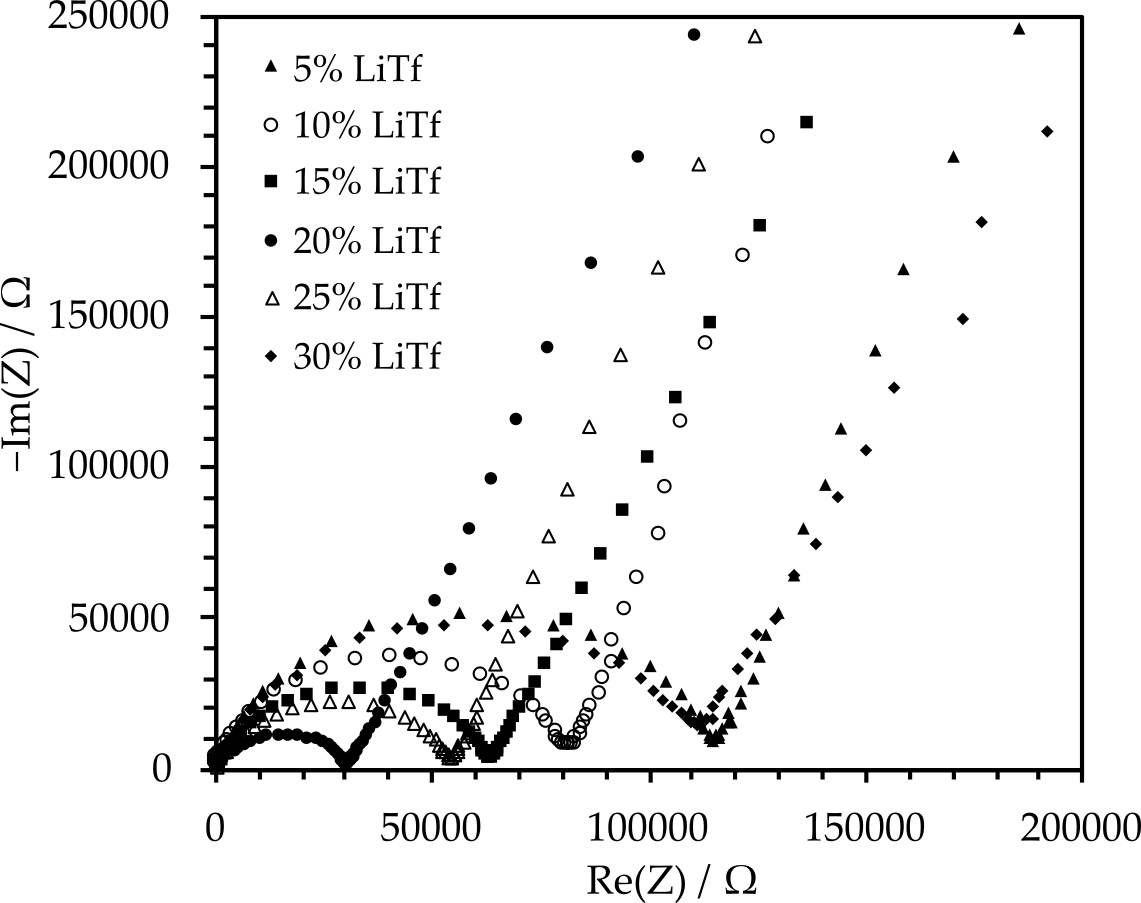

Supplement: Supplementary file 1 [file molecules-29-04526-s001.zip › molecules-3198227-supplementary/Figure S3.tiff]

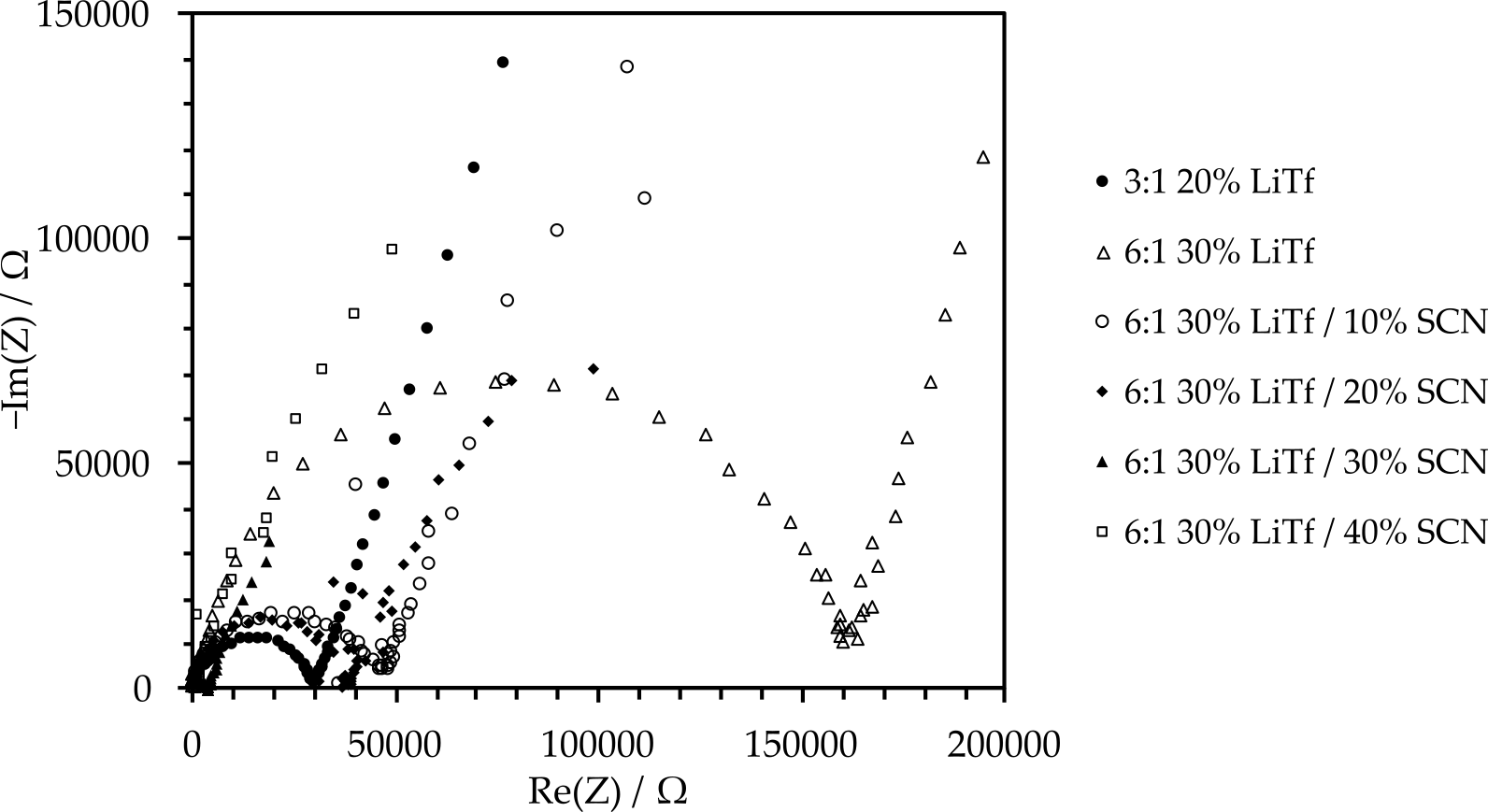

Supplement: Supplementary file 1 [file molecules-29-04526-s001.zip › molecules-3198227-supplementary/Figure S4.tiff]

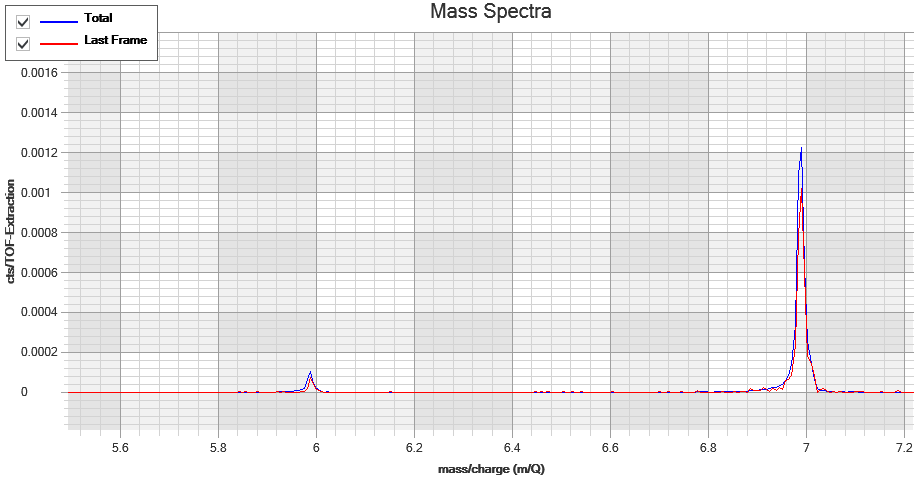

Supplement: Supplementary file 1 [file molecules-29-04526-s001.zip › molecules-3198227-supplementary/Figure S5.png]

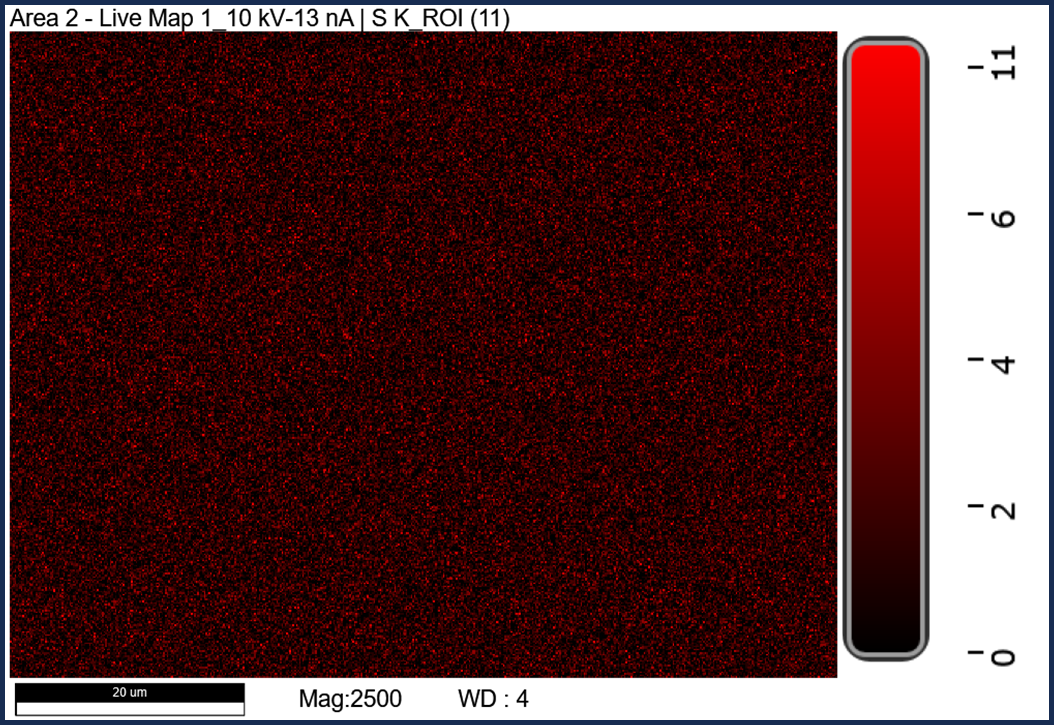

Supplement: Supplementary file 1 [file molecules-29-04526-s001.zip › molecules-3198227-supplementary/Figure S6.tif]

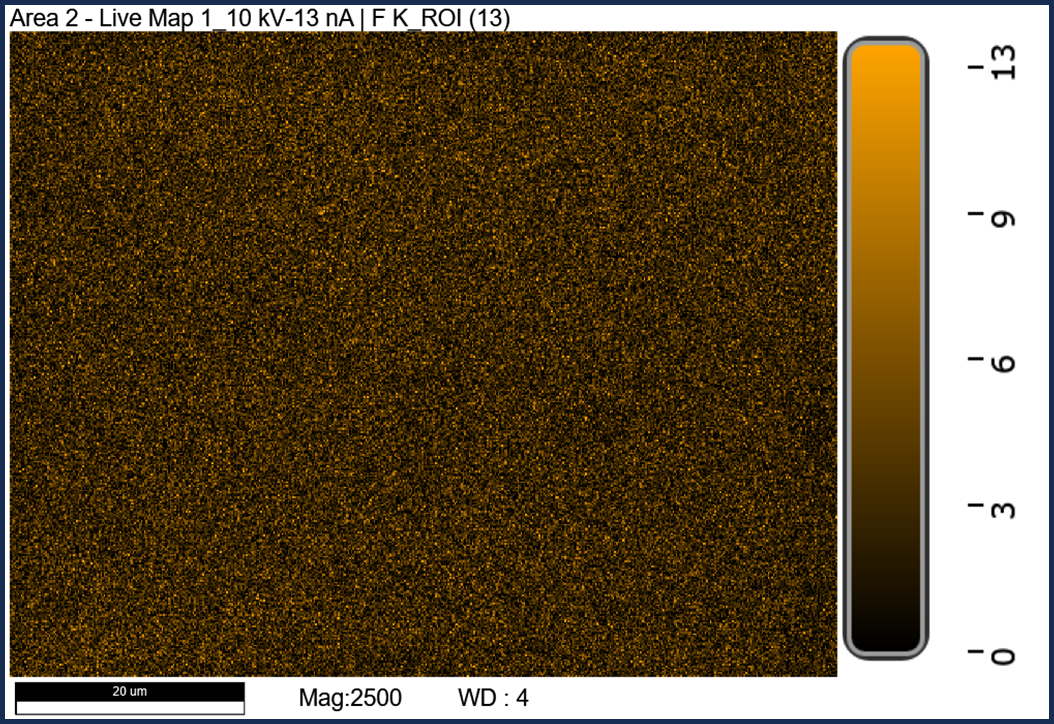

Supplement: Supplementary file 1 [file molecules-29-04526-s001.zip › molecules-3198227-supplementary/Figure S7.tif]

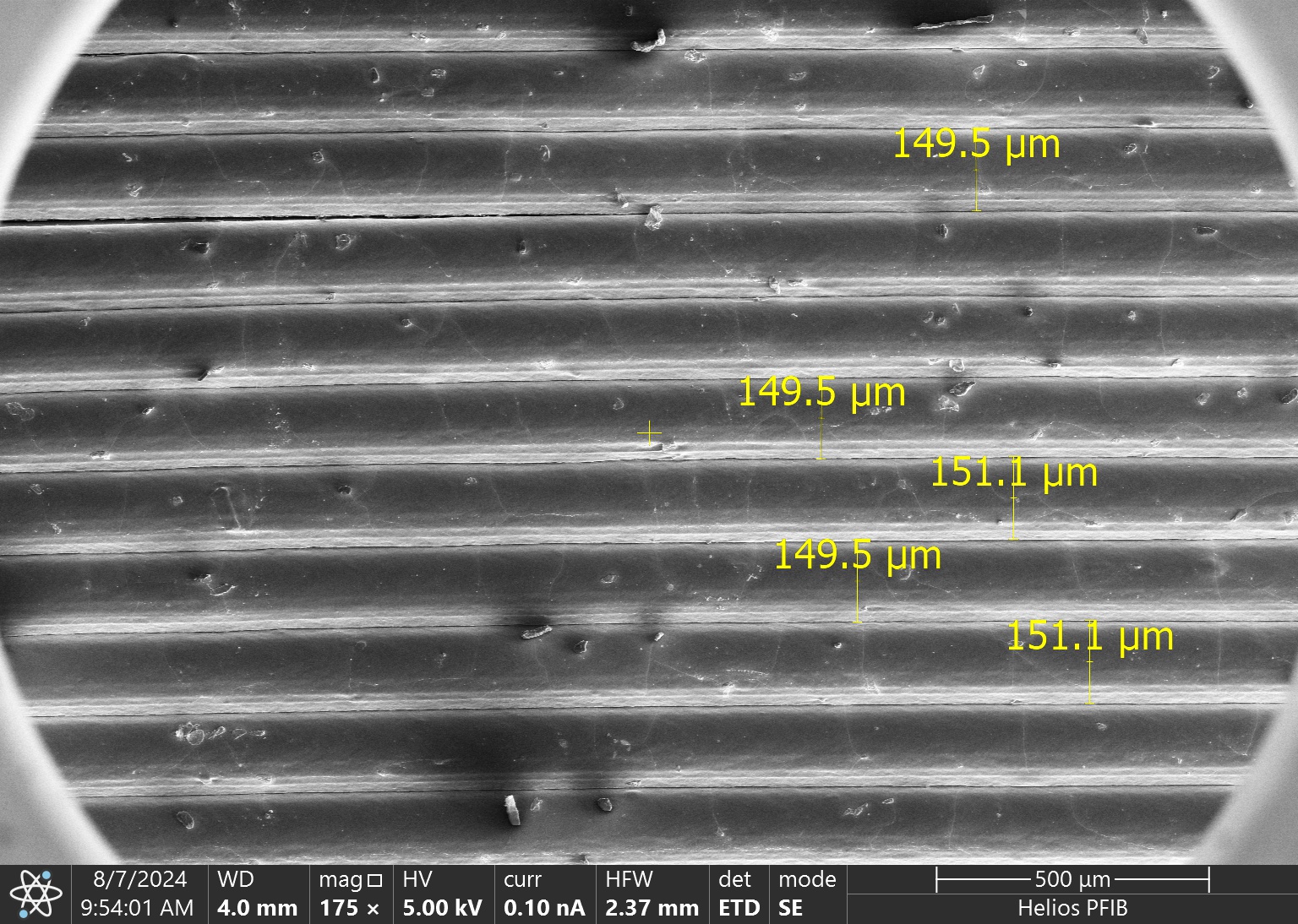

Supplement: Supplementary file 1 [file molecules-29-04526-s001.zip › molecules-3198227-supplementary/Figure S8.jpg]
